# Associated characteristics and treatment outcomes of medication-related osteonecrosis of the jaw in patients receiving denosumab or zoledronic acid for bone metastases

**DOI:** 10.1007/s00520-021-06018-x

**Published:** 2021-02-01

**Authors:** Hiroaki Ikesue, Moe Mouri, Hideaki Tomita, Masaki Hirabatake, Mai Ikemura, Nobuyuki Muroi, Shinsuke Yamamoto, Toshihiko Takenobu, Keisuke Tomii, Mutsushi Kawakita, Hironori Katoh, Takayuki Ishikawa, Hisateru Yasui, Tohru Hashida

**Affiliations:** 1 Department of Pharmacy, Kobe City Medical Center General Hospital, 2-2-1, Minatojima-Minamimachi, Chuo-ku, Kobe, 650-0047 Japan; 2 Graduate School of Pharmaceutical Sciences, Kobe Gakuin University, 1-1-3 Minatojima, Chuo-ku, Kobe, 650-8586 Japan; 3 Present Address: Department of Pharmacy, Kishiwada City Hospital, 1001, Gakuhara-cho, Kishiwada-shi, Osaka, 596-8501 Japan; 4 Division of Education and Research Promotion for Clinical Pharmacy, Faculty of Pharmaceutical Sciences, Kobe Gakuin University, 1-1-3 Minatojima, Chuo-ku, Kobe, 650-8586 Japan; 5 Department of Oral and Maxillofacial Surgery, Kobe City Medical Center General Hospital, Kobe, Japan; 6 Department of Respiratory Medicine, Kobe City Medical Center General Hospital, Kobe, Japan; 7 Department of Urology, Kobe City Medical Center General Hospital, Kobe, Japan; 8 Department of Breast Surgery, Kobe City Medical Center General Hospital, Kobe, Japan; 9 Department of Hematology, Kobe City Medical Center General Hospital, Kobe, Japan; 10 Department of Medical Oncology, Kobe City Medical Center General Hospital, Kobe, Japan

**Keywords:** Denosumab, Zoledronic acid, Osteonecrosis of the jaw, Oral health status, Resolution

## Abstract

**Purpose:**

This study aimed to evaluate the association between clinical characteristics and development of medication-related osteonecrosis of the jaw (MRONJ) in patients who underwent dental examinations before the initiation of treatment with denosumab or zoledronic acid, which are bone-modifying agents (BMAs), for bone metastases. Additionally, the clinical outcomes of patients who developed MRONJ were evaluated along with the time to resolution of MRONJ.

**Methods:**

The medical charts of patients with cancer who received denosumab or zoledronic acid for bone metastases between January 2012 and September 2016 were retrospectively reviewed. Patients were excluded if they did not undergo a dental examination at baseline.

**Results:**

Among the 374 included patients, 34 (9.1%) developed MRONJ. The incidence of MRONJ was significantly higher in the denosumab group than in the zoledronic acid (27/215 [12.6%] vs 7/159 [4.4%], *P* = 0.006) group. Multivariate Cox proportional hazards regression analysis revealed that denosumab treatment, older age, and tooth extraction before and after starting BMA treatments were significantly associated with developing MRONJ. The time to resolution of MRONJ was significantly shorter for patients who received denosumab (median 26.8 months) than for those who received zoledronic acid (median not reached; *P* = 0.024).

**Conclusion:**

The results of this study suggest that treatment with denosumab, age > 65 years, and tooth extraction before and after starting BMA treatments are significantly associated with developing MRONJ in patients undergoing treatment for bone metastases. However, MRONJ caused by denosumab resolves faster than that caused by zoledronic acid.

## Introduction

Bone metastases are common in advanced cancer, resulting in clinically important complications such as cancer-related pain, fractures, spinal cord compression, and hypercalcemia [1]. Although the direct influence of skeletal-related events (SREs) on the prognosis of advanced cancer may be limited, SREs remarkably decrease the quality of life for these patients [2]. Cancer therapy has prolonged the survival for patients with advanced cancer [3, 4]. Therefore, the prevalence of bone metastases from cancer has inevitably increased, with an accompanying increase in the significance of treatment [5, 6].

Bisphosphonates (BPs), which have a high chemical affinity for bone and specifically inhibit osteoclastic bone resorption, have been widely used for the treatment of bone metastasis. Zoledronic acid exhibits greater potency than other BPs used in several preclinical models of bone resorption [7]. Furthermore, BPs have been shown to decrease and/or delay the onset of SREs and reduce tumor-induced hypercalcemia and bone pain [8–10]. Denosumab is a fully humanized monoclonal antibody with high affinity and specificity for nuclear factor-kappa-B (NFκB) ligand (RANKL) [11]. The results of randomized controlled trials comparing denosumab and zoledronic acid for the prevention of SREs in metastatic bone diseases have shown that denosumab is superior in cases of breast [12] and prostate cancer [13] and noninferior in cases of solid tumors and multiple myeloma [14, 15]. Both zoledronic acid and denosumab are widely used for the treatment of bone metastases.

Although the effectiveness of bone-modifying agents (BMAs) in the treatment of bone metastases due to cancer has been established, medication-related osteonecrosis of the jaw (MRONJ) is known to be a significant adverse event associated with the use of BMAs since the first report in 2003 [16, 17]. MRONJ causes significant pain and reduces the quality of life; therefore, multidisciplinary team care that enables appropriate monitoring and referral to a dental specialist for close follow-up and assessment of early-stage MRONJ is recommended [18–21]. Several risk factors for MRONJ have been reported including medication-related risk factors (such as BMAs, antiangiogenic agents, systemic steroids, and time of exposure of those medications), patient-related risk factors (older age, diabetes mellitus, smoking), and oral health-related risk factors (oral infections and periodontal disease, poor oral health, implants, tooth extractions, and dentoalveolar surgery before and during the treatment) [18–23]. Dental examination before the initiation of treatment with BMAs is recommended for minimizing the risk of MRONJ [18–21, 24]. Even though the majority of patients in our hospital undergo dental examination before the initiation of BMA treatment and underwent dental procedures including tooth extraction before the initiation of BMAs when necessary, some still develop MRONJ. In several previous studies regarding the risk of developing MRONJ [25–31], the study subjects included patients who underwent dental examinations before initiation of BMA treatment as well as those who received BMAs without dental examination. However, the precise risk factors for MRONJ in patients who undergo dental examinations before initiation of BMA treatment remain unclear [24, 31].

Therefore, the aim of this study was to evaluate the association between clinical characteristics and MRONJ development in patients who had undergone dental examinations, and dental procedures including tooth extraction when necessary, before treatment initiation with denosumab or zoledronic acid, which are bone-modifying agents (BMAs), for bone metastases. In addition, the clinical outcomes of patients who developed MRONJ were evaluated along with the time to resolution of MRONJ.

## Patients and methods

### Study design, setting, and participants

We conducted a retrospective cohort study, where we reviewed the medical records of patients with cancer who received denosumab or zoledronic acid for bone metastases after approval by a dentist between January 2012 and September 2016. This study was conducted in accordance with the Declaration of Helsinki. The study protocol was approved by the Ethics Committee of Kobe City Medical Center General Hospital (approval numbers: zn171010 and k181010). Patients treated with denosumab and zoledronic acid were identified from an electronic medical and dental record system in our hospital. Patients were eligible if they were ≥ 20 years of age, diagnosed with solid tumors or multiple myeloma, had at least one bone metastasis or osteolytic lesion, and received denosumab or zoledronic acid treatment at any of the following five departments in Kobe City Medical Center General Hospital between January 1, 2012, and September 30, 2016: Department of Respiratory Medicine, Department of Urology, Department of Breast Surgery, Department of Hematology, and Department of Medical Oncology. The exclusion criteria were as follows: no dental examination before the initiation of denosumab or zoledronic acid treatment, use of zoledronic acid for the treatment of hypercalcemia, lack of follow-up for at least 1 month after the treatment, history of radiation therapy of the jaws, and treatment with both denosumab and zoledronic acid.

### Treatment procedure for bone metastases

Following dental examination, when needed, patients underwent dental procedures including tooth extraction to minimize the risk of developing MRONJ before the initiation of BMAs. All patients were administered denosumab 120 mg subcutaneously every 4 weeks or zoledronic acid 4 mg intravenously every 3 to 4 weeks. Patients with impaired kidney function (creatinine clearance of ≤ 60 mL/min) were given a manufacturer-recommended reduced dose of zoledronic acid (3–3.5 mg) according to the same administration schedule as that for patients with normal kidney function.

### Data source and variables

We collected data from electronic medical and dental records including sex, age, weight, type of cancer, comorbidities, concomitant medications, antiresorptive therapies, number of treatment courses, tooth extraction before and after starting BMA treatments, MRONJ stage, MRONJ treatment, and outcomes. To reduce the potential bias for evaluating patient and treatment characteristics associated with developing MRONJ, we limited study participants to those examined by dentists before starting BMA treatments, because previous studies reported that poor oral health status was a significant risk factor for developing MRONJ [17, 19, 21, 24]. Furthermore, all patients were recommended to visit dental clinics routinely after BMA initiation. If the patients were considering invasive dental procedures including tooth extraction after the beginning of BMAs, those patients were asked to consult with dentists in our hospital. After initiation of BMA treatments, patients who complained of dental symptoms such as pain or oral discomfort consulted with a dentist following the attending physician’s request. Tooth extraction was performed in unavoidable situations including accidental root fracture or acute exacerbation of periodontal disease. MRONJ diagnosis was determined from clinical and radiographical findings. MRONJ was diagnosed by dentists in our hospital according to the criteria stated in the American Association of Oral and Maxillofacial Surgeons (AAOMS) position paper [17].

### Treatments and outcomes of MRONJ

The treatment methods were divided into two categories: (i) conservative measures, including the use of an antiseptic mouth rinse, systemic antimicrobial agent, and/or debridement of bony sequestra separated from the surface of the exposed bone, and (ii) surgical treatment [32]. After MRONJ diagnosis, osteonecrotic regions were observed with conservative treatment such as antibiotics. Subsequently, if a bony sequestrum was formed in the osteonecrotic region, it was surgically removed. Surgical treatment involved conservative surgery, which was defined as the removal of only necrotic bone, or extensive surgery, defined as the removal of necrotic and surrounding healthy bone, i.e., marginal mandibulectomy or partial maxillectomy. In our institution, surgical treatment was usually conducted after conservative measures for a certain period.

The clinical outcomes of MRONJ were also evaluated by dentists in our hospital according to the position paper laid down by the AAOMS [17], and then revised according to the guidelines by the Multinational Association of Supportive Care in Cancer (MASCC), International Society of Oral Oncology (ISOO), and American Society of Clinical Oncology (ASCO) [21]. The treatment outcome was divided into four categories: resolved (defined as complete coverage of the exposed bone by mucosa in the absence of clinical symptoms), improving, stable, and progressive [21]. The cut-off date for diagnosing MRONJ and evaluating the treatment outcomes were December 31, 2017, and July 31, 2019, respectively.

The primary end-point was the association between the clinical characteristics and development of MRONJ, whereas secondary end-points included the probability of MRONJ, the relationship between those characteristics and the time to onset of MRONJ, and the relationship between the type of antiresorptive drug and treatment outcome.

### Statistical analysis

Categorical data were compared between groups using the Chi-square test or Fisher’s exact test as appropriate. Continuous data with normal distribution are presented as mean ± standard deviation, while those without normal distribution are presented as median (interquartile range). Student’s *t* test was used to compare normally distributed variables, while the Mann–Whitney *U* test was used to compare variables without normality. Univariate and multivariate Cox proportional hazards regression models were used for identifying the potential significant factors influencing MRONJ development. Variables with a *P* value of <0.05 in the univariate analyses were evaluated as potential covariates in the multivariate analysis. The time to development of MRONJ and the time to MRONJ resolution were compared between the groups using the Kaplan–Meier method with the log-rank test. All statistical analyses were performed using JMP 13.0.0 (SAS Institute Inc., Cary NC, USA). A *P* value of < 0.05 was considered statistically significant.

## Results

### Patient characteristics

**Fig. 1:**
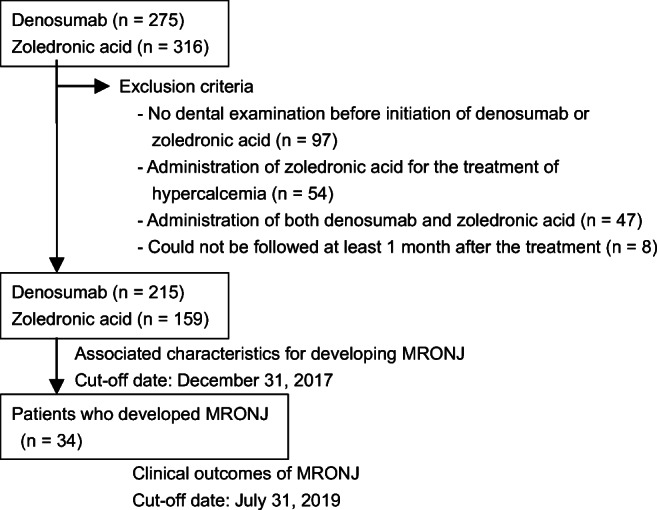
Study flowchart. MRONJ, medication-related osteonecrosis of the jaw

Between January 2012 and September 2016, 580 adult patients with bone metastases due to cancer were treated with denosumab or zoledronic acid. Among these, 206 patients were excluded because they did not undergo dental examinations before the initiation of treatment with denosumab or zoledronic acid (*n* = 97), received zoledronic acid for the treatment of hypercalcemia (*n* = 54), received both denosumab and zoledronic acid (*n* = 47), or could not be followed up for at least 1 month following treatment (*n* = 8) (Fig. 1). For the remaining 374 patients (215 in the denosumab group and 159 in the zoledronic acid group), the median follow-up duration was 15.5 months (IQR 12.2–21.3). The median (IQR) follow-up time was significantly longer in the zoledronic acid group than in the denosumab group (16.8 [13.2–26.4] vs 14.6 [11.7–19.5] months, *P* <0.001). In total, 34 patients (9.1%) developed MRONJ. The patient characteristics are shown in Table 1. The incidence of MRONJ was significantly higher in the denosumab group than in the zoledronic acid group (12.6 vs 4.4%, *P* = 0.006). The median (IQR) number of treatment courses was significantly longer for patients who developed MRONJ than for those who did not (16 [12–24] vs 6 [2–11], *P* < 0.001).

**Table 1:** Baseline characteristics of patients who developed MRONJ or not

Characteristics	Patients with MRONJ (*n* = 34)	Patients without MRONJ (*n* = 340)	*P* value
Male sex, *n* (%)	20 (58.8%)	192 (56.5%)	0.857
Age (years), median (IQR)	70 (65–77)	68 (62–75)	0.191
Weight (kg), median (IQR)	56.8 (50.6–62.6)	54.3 (46.5–62.4)	0.271
Type of disease, *n* (%)			–
Lung cancer	10 (29.4%)	143 (42.3%)	
Breast cancer	6 (17.7%)	61 (18.1%)	
Multiple myeloma	4 (11.8%)	52 (15.4%)	
Prostate cancer	12 (35.3%)	42 (12.4%)	
Others^a^	2 (5.9%)	40 (11.8%)	
Comorbid disease, *n* (%)			
Hypertension	16 (47.1%)	140 (41.2%)	0.585
Diabetes	3 (8.8%)	67 (19.7%)	0.165
Tooth extraction before starting BMAs, *n* (%)	18 (51.4%)	75 (22.1%)	< 0.001
Concomitant medication, *n* (%)			
Oral bisphosphonate	1 (2.9%)	12 (3.5%)	1.000
Antiangiogenic agents ^b^	11 (32.4%)	62 (18.2%)	0.067
Steroid	22 (64.7%)	181 (53.2%)	0.212
Treatment agents, *n* (%)			
Denosumab	27 (79.4%)	188 (55.3%)	0.006
Zoledronic acid	7 (20.6%)	152 (44.7%)	
Number of treatment courses, median (IQR)			
Denosumab	15 (10–27)	8 (3–19)	0.034
Zoledronic acid	17 (16–23)	6 (2–15)	0.001
Tooth extraction after starting BMAs, *n* (%)	10 (28.6%)	10 (2.9%)	< 0.001

For continuous values, data are presented as the median (interquartile range (IQR))

*MRONJ*, medication-related osteonecrosis of the jaw

^a^Includes renal cell carcinoma (*n* = 2) in patients with MRONJ, renal cell carcinoma (*n* = 13), gastric cancer (*n* = 5), colorectal cancer (*n* = 5), bladder cancer (*n* = 5), pancreatic cancer (*n* = 4), hepatocellular cancer (*n* = 3), esophageal cancer (*n* = 2), pharyngeal cancer (*n* = 1), extra mammary Paget’s disease (*n* = 1), and cancer of unknown primary (*n* = 1) in patients without MRONJ

^b^Includes axitinib, bevacizumab, everolimus, pazopanib, ramucirumab, regorafenib, sorafenib, sunitinib, and temsirolimus

Among patients in the denosumab group, the incidence of MRONJ in patients with lung cancer, breast cancer, multiple myeloma, prostate cancer, and other type of cancers was 8.9% (10/113), 13.5% (7/52), 100% (1/1), 26.3% (10/38), and 0% (0/9), respectively. Among the zoledronic acid group, the incidence of MRONJ in patients with lung cancer, breast cancer, multiple myeloma, prostate cancer, and other type of cancers was 0% (0/40), 0% (0/15), 5.5% (3/55), 12.5% (2/33), and 6.1% (2/33), respectively. In addition, the median number of treatment courses (12 vs 6 times, *P* <0.001) and median age (74 vs 67 years old, *P* <0.001) were both significantly higher in patients with prostate cancer than in patients with other cancer types.

The differences in patient characteristics between the denosumab and zoledronic acid groups are shown in Table 2. The distribution of cancer types was significantly different between groups (*P* <0.001).

**Table 2:** Baseline characteristics of patients between denosumab and zoledronic acid groups

Characteristics	Denosumab (*n* = 215)	Zoledronic acid (*n* = 159)	*P* value
Male sex, *n* (%)	115 (53.5%)	97 (61.0%)	0.170
Age (years), median (IQR)	68 (61–75)	69 (63–76)	0.486
Weight (kg), median (IQR)	56.0 (48.8–62.5)	54.0 (45.2–62.0)	0.192
Type of disease, *n* (%)			
Lung cancer	113 (53.1%)	40 (25.2%)	<0.001
Breast cancer	52 (24.4%)	15 (9.4%)	
Multiple myeloma	1 (0.5%)	55 (34.6%)	
Prostate cancer	38 (17.8%)	16 (10.1%)	
Others	9 (4.2%)	33 (20.8%)	
Comorbid disease, *n* (%)			
Hypertension	84 (39.1%)	72 (45.3%)	0.244
Diabetes	40 (18.6%)	30 (18.9%)	1.000
Tooth extraction before starting BMAs, *n* (%)	58 (27.0%)	35 (22.0%)	0.279
Concomitant medication, *n* (%)			
Oral bisphosphonate	6 (2.8%)	7 (4.4%)	0.409
Antiangiogenic agents ^a^	47 (21.9%)	26 (16.4%)	0.190
Steroid	105 (48.8%)	61 (38.4%)	0.016
Tooth extraction after starting BMAs, *n* (%)	13 (6.1%)	7 (4.4%)	0.643

For continuous values, data are presented as the median (interquartile range (IQR))

*MRONJ*, medication-related osteonecrosis of the jaw

^a^Includes axitinib, bevacizumab, everolimus, pazopanib, ramucirumab, regorafenib, sorafenib, sunitinib, and temsirolimus

### Association between clinical characteristics and MRONJ development

**Fig. 2:**
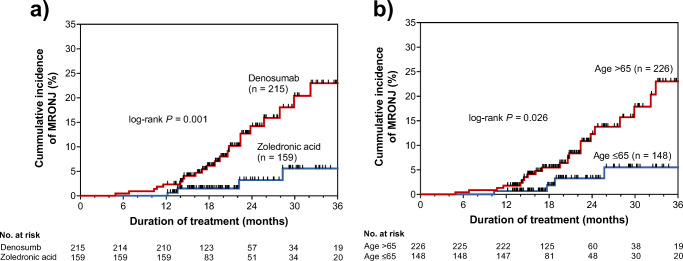
Cumulative incidence of medication-related osteonecrosis of the jaw in patients receiving denosumab or zoledronic acid for bone metastases. MRONJ, medication-related osteonecrosis of the jaw

The univariate analyses showed that treatment with denosumab (hazards ratio [HR], 4.28; 95% confidence interval [CI], 1.81–11.86; *P* = 0.001), older age (>65 years; HR, 2.43; 95% CI, 1.10–6.13; *P* = 0.028), tooth extraction before starting BMAs (HR, 3.52; 95% CI, 1.70–7.44; *P* = 0.001), and tooth extraction after starting BMAs (HR, 3.74; 95% CI, 1.51–8.42; *P* = 0.006) were significantly associated with the development of MRONJ in patients receiving denosumab or zoledronic acid (Table 3). Subsequently, the multivariate Cox proportional hazards regression analysis also showed that treatment with denosumab (HR, 6.53; 95% CI, 2.62–19.12; *P* < 0.001), older age (>65 years; HR, 3.34; 95% CI, 1.46–8.68; *P* = 0.004), tooth extraction before starting BMAs (HR, 3.52; 95% CI, 1.70–7.44; *P* = 0.001), and tooth extraction thereafter (HR, 3.74; 95% CI, 1.51–8.42; *P* = 0.006) were significantly associated with developing MRONJ. To further explore the relationship between these characteristics and MRONJ development, we analyzed the time to onset of MRONJ using Kaplan–Meier analysis (Fig. 2). The cumulative incidence of MRONJ was significantly higher in the denosumab group than in the zoledronic acid group (*P* = 0.001). Similarly, patients aged ≥65 years (*P* = 0.034) showed a significantly higher cumulative incidence of MRONJ.

**Table 3:** Univariate and multivariate analyses of association between clinical characteristics and developing for medication-related osteonecrosis of the jaw in patients who received denosumab or zoledronic acid for bone metastases

Variables	Univariate analyses			Multivariate analysis		
	Hazard ratio	95% CI	*P* value	Hazard ratio	95% CI	*P* value
Denosumab treatment	4.28	1.81–11.86	0.001	6.53	2.62–19.12	< 0.001
Age (> 65 years)	2.43	1.10–6.15	0.028	3.34	1.46–8.68	0.004
Tooth extraction before starting BMAs	3.06	1.50–6.33	0.002	3.52	1.70–7.44	0.001
Tooth extraction after starting BMAs	3.82	1.61–8.39	0.004	3.74	1.51–8.42	0.006
Male sex	1.03	0.50–2.17	0.936	N/A		
Weight (kg)	0.99	0.96–1.03	0.743	N/A		
Hypertension	1.27	0.61–2.63	0.513	N/A		
Diabetes	0.49	0.12–1.40	0.201	N/A		
Concomitant use of antiangiogenic agents ^a^	1.35	0.59–2.89	0.460	N/A		
Concomitant use of steroids	1.37	0.66–2.98	0.405	N/A		

*CI* confidence interval, *BMA* bone-modifying agent

N/A indicates that the covariate was not included in the model because it was not significant in univariate analyses

^a^Includes axitinib, bevacizumab, everolimus, pazopanib, ramucirumab, regorafenib, sorafenib, sunitinib, and temsirolimus

### Treatment and outcomes

**Fig. 3:**
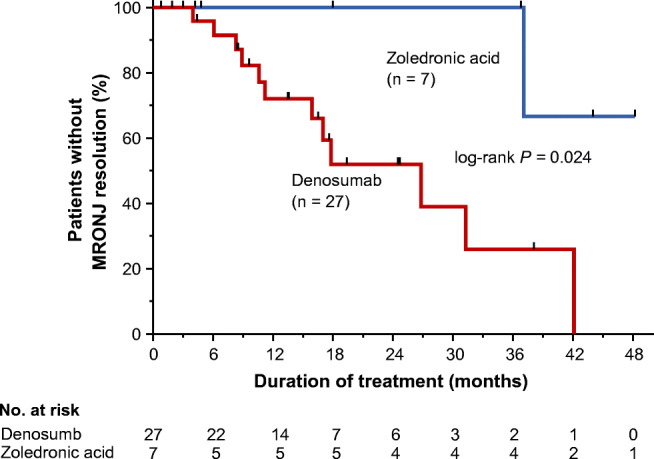
Time to resolution of denosumab- or zoledronic acid-related osteonecrosis of the jaw in patients with bone metastases. A total of 34 patients who developed MRONJ were evaluated. MRONJ, medication-related osteonecrosis of the jaw

The characteristics and treatment of the 34 patients who developed MRONJ are summarized in Table 4. The median follow-up period was 14.8 months (IQR 7.8–25.2). All these patients were treated by dentists, and BMAs were discontinued after careful discussion between dentists and physicians, in 88.9% (24/27) of patient receiving denosumab and 100% (7/7) of patients receiving zoledronic acid. At the final follow-up, MRONJ had resolved in 12 (44.4%) and one (14.3%), improved in six (22.2%) and zero (0%), and was stable/progressive in 9 (33.3%) and six (85.7%) patients being treated with denosumab and zoledronic acid, respectively. The median time from MRONJ onset to resolution was 31.3 months. As shown in Fig. 3, the time to MRONJ resolution was significantly shorter in the denosumab group (median 26.8 months) than in the zoledronic acid group (median not reached; *P* = 0.024).

**Table 4:** Patient characteristics and treatment with denosumab- or zoledronic acid-related osteonecrosis of the jaw

	Denosumab (*n* = 27)	Zoledronic acid (*n* = 7)
Male sex, *n* (%)	15 (53.6%)	5 (71.4%)
Age (years), median (IQR)	71 (65–78)	69 (65–77)
Weight (kg), median (IQR)	56.6 (49.4–61.9)	57.8 (51.2–68.9)
Type of disease, *n* (%)		
Lung cancer	10 (35.7%)	0 (0%)
Breast cancer	7 (25.0%)	0 (0%)
Multiple myeloma	1 (3.6%)	3 (42.9%)
Prostate cancer	10 (35.7%)	2 (28.6%)
Others	0 (0%)	2 (28.6%)
Comorbid disease, *n* (%)		
Hypertension	12 (42.9%)	4 (57.1%)
Diabetes	3 (10.7%)	0 (0%)
Tooth extraction before starting BMAs, *n* (%)	14 (50.0%)	4 (57.0%)
Concomitant medication, *n* (%)		
Antiangiogenic agents ^a^	9 (21.9%)	26 (16.4%)
Steroid	105 (48.8%)	61 (38.4%)
Tooth extraction after starting BMAs, *n* (%)	13 (6.1%)	7 (4.4%)
Time to onset of MRONJ (months)	18.8 (11.6–25.7)	32.9 (19.1–43.3)
Stage		
0	1 (3.7%)	0 (0%)
1	7 (22.2%)	1 (14.3%)
2	19 (70.4%)	5 (71.4%)
3	1 (3.7%)	1 (14.3%)
Affected jaw		
Mandible	19 (70.4%)	3 (42.9%)
Maxilla	7 (25.9%)	4 (57.1%)
Mandible and maxilla	1 (3.7%)	0 (0%)
Discontinuation of BMAs	24 (88.9%)	7 (100%)
Treatment		
Conservative measures	9 (33.3%)	4 (57.1%)
Conservative surgery ^b^	14 (51.9%)	2 (28.6%)
Extensive surgery ^b^	4 (14.8%)	1 (14.3%)

For continuous values, data are presented as the median (interquartile range (IQR))

*BMA* bone-modifying agent, *MRONJ* medication-related osteonecrosis of the jaw

^a^Includes axitinib, bevacizumab, everolimus, pazopanib, ramucirumab, regorafenib, sorafenib, sunitinib, and temsirolimus

^b^Conservative or extensive surgery was conducted after conservative measures in most cases

There were 23 patients with sequestration (20 and 3 patients in the denosumab and zoledronic acid group, respectively). The sequestrum was removed by dentists in 21 patients and spontaneously cast-off in two. Of these 21 patients, 16 are categorized as conservative surgery in Table 4, and five categorized as extensive surgery because they underwent other concomitant extensive procedures. The outcome was resolved in 11 and improving in five patients. Among five residual patients, in three, the outcomes were still observed after the removal procedure, but two could not be evaluated because they moved to another hospital soon after the removal. The median time between MRONJ onset to sequestration surgery was 9.9 months.

## Discussion

To date, information regarding the precise risk factors for MRONJ in patients who receive dental examinations before BMA treatment initiation is scarce [24, 31]. The present study clearly showed that treatment with denosumab and older age (>65 years) were significantly associated with developing MRONJ in patients who received dental examinations before treatment with denosumab or zoledronic acid for bone metastases. Although denosumab significantly increased the development of MRONJ, the time to resolution was significantly shorter in the denosumab group than in the zoledronic acid group.

The reported incidence of MRONJ is 1–17% [12–15, 20, 24, 26, 29, 31, 33–37]. The incidence of MRONJ in the present study was within this range in both the denosumab (12.6%) and zoledronic acid (4.4%) groups. The multivariate analysis revealed that treatment with denosumab had a significantly higher risk of developing MRONJ than treatment with zoledronic acid. On the other hand, previous randomized controlled trials found that the incidence of MRONJ in patients treated with denosumab was not significantly different from that in patients treated with zoledronic acid, although it tended to be higher [13, 33, 36]. In our study, data from real-word clinical practice, vast majority of the study subjects were not had scheduled periodic dental examinations, and if the patients complained of dental symptoms, the attending physicians consulted to the dentists. On the other hand, in randomized controlled trials, the protocol had specified that all the participants underwent scheduled periodic dental examinations (e.g., at baseline and every 6 months thereafter) [12–14, 33]. Because scheduled dental examination can reduce the risk of developing MRONJ, this discordance may affect to reduce the risk of MRONJ in clinical trials. In fact, some real-world data has shown that the risk of MRONJ is increased in patients treated with denosumab [20, 38]. Inhibition of osteoclast function seems to be part of the pathophysiology of MRONJ, because the agents most commonly linked to MRONJ, namely BPs and denosumab, both reduce bone resorption, albeit via different mechanisms [17–19]. The higher incidence of denosumab-associated ONJ suggests that the effect of denosumab on bone resorption is superior to that of zoledronic acid [12, 13]. The other characteristics significantly associated with developing MRONJ in this study were older age (>65 years), and tooth extraction before and after starting BMAs. These results are consistent with the findings of previous reports [17–23]. Since tooth extractions just before starting BMAs did not reduce the risk of developing MRONJ in this study, earlier dental consultation and dental treatment should be considered after patients are diagnosed with cancer.

Other factors have been reported to elevate the risk of developing MRONJ such as concomitant use of antiangiogenic agents [18–21]. However, in this study, antiangiogenic agents did not significantly affect the incidence of MRONJ. We do not know the reason behind these results, but the effect of these medications should be investigated with more detail in future studies.

In patients with prostate cancer, the incidence of MRONJ tended to be higher than in patients with other types of cancers. Since the median number of treatment courses and median age were both significantly higher in patients with prostate cancer, we speculated that the higher incidence of MRONJ in patients with prostate cancer may be due to these differences in patient characteristics.

Interestingly, the time to MRONJ resolution was significantly shorter for patients treated with denosumab than for those treated with zoledronic acid. Theoretically, MRONJ may resolve more rapidly after drug discontinuation in patients receiving denosumab than in patients receiving BP. Since denosumab has a reversible effect on RANKL, osteoclast inhibition may reverse more quickly and allow for more rapid resolution of MRONJ relative to that in patients receiving BPs, which accumulate in the bone matrix and prolong osteoclast inhibition. The reversibility of denosumab-related ONJ is supported in a study by de Molon et al. [39], which assessed animal models of MRONJ. In addition, Saad et al. [33] analyzed the results of three phase III trials including patients with bone metastases from cancer and found that the rate of resolution of MRONJ was higher for patients taking denosumab (40%) than for those taking zoledronic acid (29%), with more rapid recovery in the former group. The authors suggested that the more rapid recovery is related to the reversible inhibition of RANKL. Our study is the first to demonstrate that the time to MRONJ resolution was significantly shorter in patients treated with denosumab than in those treated with zoledronic acid. However, it should be noted that although all MRONJ cases in this study were diagnosed by dentists in our hospital based on clinical and radiographical findings, computed tomography was not employed in all cases during diagnosis, staging, and especially by follow-up of MRONJ. Since the possibility of under evaluation for MRONJ could not be completely excluded, our preliminary results, especially relating to the assessment of resolution, should be confirmed in further studies.

One of the limitations of our study was the small number of patients and that it was conducted in a single center. Oral health status such as periodontal diseases, dental prosthesis, dental implants, and periodontal surgeries were also not fully investigated in this study. In addition, since the patients who complained of dental symptoms consulted with a dentist following the attending physician’s request, mild cases of MRONJ might be underdiagnosed. Lastly, the total number of MRONJ cases was limited, and BMAs were discontinued in most patients who developed MRONJ in our study. Therefore, the results pertaining to MRONJ resolution should be considered as preliminary data. Despite these limitations, this real-world observational study demonstrated, for the first time, that MRONJ resolved more rapidly in patients treated with denosumab than in those treated with zoledronic acid.

## Conclusions

The results of this study suggest that denosumab treatment, age >65 years, and tooth extraction before and after starting BMA treatments are significantly associated with developing MRONJ in patients undergoing treatment for bone metastases. However, MRONJ caused by denosumab resolves faster than that caused by zoledronic acid.

## Data Availability

All data generated or analyzed during this study are included in this article.
